# Evaluation of a multiantigen mRNA vaccine against EEHV1A in Asian elephants

**DOI:** 10.1128/jvi.00388-26

**Published:** 2026-07-28

**Authors:** Lauren Farris, Jessica Watts, Jeroen Pollet, Christine Molter, Sarah Cannizzo, Michael Wenninger, Jessica Heinz, Rebecca Eddy, Jessica Emerson, Dewey Maddox, Paul D. Ling

**Affiliations:** 1 Department of Molecular Virology and Microbiology, Baylor College of Medicine 3989, Houston, Texas, USA; 2 Department of Pediatrics, Division of Tropical Medicine, Baylor College of Medicine 3989, Houston, Texas, USA; 3 National School of Tropical Medicine, Baylor College of Medicine 3989, Houston, Texas, USA; 4 Houston Zoo, Inc. 374061 , Houston, Texas, USA; 5 Fort Worth Zoo, Fort Worth, Texas, USA; 6 Cincinnati Zoo and Botanical Garden 57701, Cincinnati, Ohio, USA; 7 Rosamond Gifford Zoo 527814 , Syracuse, New York, USA; 8 White Oak Conservation Center, Yulee, Florida, USA; Dartmouth College Geisel School of Medicine, Hanover, New Hampshire, USA

**Keywords:** elephant endotheliotropic herpesvirus, vaccine, herpesvirus, elephant

## Abstract

Elephant endotheliotropic herpesvirus (EEHV) is endemic in Asian elephants and can cause fatal hemorrhagic disease (HD) in juveniles, with calves aged 1–8 years being most vulnerable, particularly following the decline of maternal antibodies. Current supportive treatments, which commonly include fluids, plasma infusions, and human anti-herpesvirus drugs, are not consistently effective, highlighting the urgent need for preventative strategies. To address this challenge, we generated an EEHV1A multi-antigen mRNA vaccine targeting four conserved herpesvirus entry proteins (gB, gH, gL, and gO), which elicited robust humoral and cellular immune responses in mice without adverse effects. Based on these preclinical findings, a vaccine trial was conducted in nine Asian elephants, comprising three seropositive and six seronegative individuals. Animals received up to four 400 μg doses intramuscularly 3 weeks apart, with serum samples collected at baseline (day 0), post-booster (day 42), and monthly through day 192. Seropositive elephants demonstrated anamnestic responses with increased antibody levels to all four vaccine antigens individually. Seronegative elephants responded more robustly to gH and gL versus variable responses to gB and gO. Enzyme-linked immunosorbent assays (ELISAs) detecting the gH/gL/gO trimer indicate sustained antibody level increases in both sero-positive and sero-negative groups, persisting up to 192 days post-vaccination in seropositive elephants. Notably, post-vaccine titers against the trimer in some seronegative elephants reached up to 50% of baseline levels observed in EEHV-experienced subadult and adult elephants. These findings represent a critical advancement toward an effective EEHV1A vaccine to reduce HD mortality and support Asian elephant conservation efforts.

**IMPORTANCE:**

Elephant endotheliotropic herpesvirus (EEHV) causes fatal hemorrhagic disease in 65% of infected juvenile Asian elephants, critically threatening this endangered species with fewer than 50,000 individuals remaining globally. This study demonstrates the first successful mRNA vaccine application in elephants, targeting EEHV1A glycoproteins essential for viral attachment and entry. The vaccine elicited sustained antibody responses for up to 6 months in both EEHV-naive and previously infected elephants, with post-vaccination levels in some seronegative animals reaching 50% of those in chronically infected adults. These immunological data provide a critical baseline understanding of protective immunity, as demonstrated in a companion case report where two vaccinated elephants from this cohort survived subsequent wild-type EEHV1A infection without hemorrhagic disease. This work establishes mRNA vaccination as a viable conservation strategy, providing a scalable platform adaptable for other EEHV subtypes and representing a crucial advancement toward preventing EEHV mortality in endangered elephants.

## INTRODUCTION

Asian elephant (*Elephas maximus*) populations, both in human care and in the wild, are threatened by a lethal hemorrhagic disease (HD) caused by elephant endotheliotropic herpesvirus (EEHV) (1). EEHV-HD is the largest cause of death in juvenile Asian elephants housed in European and North American institutions and has a reported fatality rate of approximately 65%. Additionally, over 100 cases have been documented in semi-captive and wild populations across Asia (1). More recently, EEHV-HD has also been identified in African Savannah elephants (*Loxodonta africana*), raising broader conservation concerns for this endangered species (2–6).

To date, seven EEHV types (EEHV1–7) have been identified, with genetically distinct subtypes (e.g., 1A and 1B) detected for almost all the types (1, 7–11). EEHV1, 4, and 5 primarily affect Asian elephants, while EEHV2, 3, 6, and 7 circulate in African elephant populations (1). Although adult elephants are typically chronically infected with multiple EEHV types, EEHV-HD is most frequently associated with a primary infection of EEHV1A and 1B in Asian elephants, and EEHV2 and 3 in African elephants (5, 6). EEHV6 has also recently been implicated in HD cases (4) and EEHV7 with clinical illness (3). The relative pathogenicity of different EEHV types in African elephants remains unclear.

Juvenile elephants are particularly vulnerable to EEHV-HD. In Asian elephants, the highest risk occurs between 1 and 8 years of age, while in African elephants, the highest risk range is somewhat wider, up to 13 years (3, 5, 6). This age-related vulnerability is thought to be linked to the waning or absence of maternal antibodies specific to the infecting EEHV type as determined by immunoreactivity to EEHV1-specific biomarkers encoded by open reading frame Q (ORFQ) or the predicted tegument proteins encoded by the E34 gene in EEHV2, 3, and 6 (5, 6, 12, 13). Similar findings have been observed using biomarkers encoded by glycoproteins gH and gL (13). Notably, cases in calves under 1 year of age are rare, likely due to the protective effect of high maternal antibody titers during early exposure.

Given the high incidence of EEHV-HD and lethality associated with EEHV1A, vaccine development efforts have focused on this subtype. Preclinical studies have demonstrated that recombinant gB-based Modified Vaccinia Ankara (MVA) and protein subunit vaccines can elicit strong antibody and T-cell responses (14, 15). Additional work has shown that a gH/gL protein subunit vaccine induces bifunctional T cells and antigen-specific antibodies in mice (16). While the immune response to gO remains less well characterized, seropositive elephants exhibit high levels of gO-specific antibodies, suggesting its potential as a vaccine target (17).

To evaluate a broader immune response, we developed a multi-antigen mRNA vaccine encoding EEHV1A gB, gH, gL, and gO representing the conserved herpesvirus attachment fusion machinery (17). In mice, this vaccine induced robust antibody responses and polyfunctional T-cell activation against all four antigens. Due to its rapid production and scalability, this mRNA platform was selected for further evaluation in elephants.

In this study, we administered an EEHV1A multi-antigen mRNA vaccine to a cohort of seropositive and seronegative Asian elephants and monitored antibody responses to the encoded proteins for up to 6 months post-vaccination. In a companion manuscript (18), we describe a case study of two of the vaccinated seronegative elephants that experienced infection from wild-type EEHV without experiencing clinical illness or detectable changes in blood chemistries or complete blood counts (CBCs), suggesting that the vaccine provided beneficial protection. These investigations represent a critical step toward developing a protective vaccine for seronegative juvenile elephants, who are at greatest risk of severe disease and death from primary EEHV1A infection.

## MATERIALS AND METHODS

### Study population and design

A cohort of nine Asian elephants housed at five zoological institutions across the United States was selected for this study. Elephants were stratified into three groups based on serostatus and vaccination timeline. Group 1 comprised three seropositive elephants. Groups 2 and 3 consisted of seronegative elephants, with Group 2 receiving vaccination approximately 5 months prior to Group 3, allowing for extended post-vaccination serological monitoring. Group 3 elephants exhibited atypical early-life histories that may influence immune development: one elephant was rejected by its dam and was bottle-fed for the first 2 years of life, while the other two were twins, an extremely rare occurrence in Asian elephants, comprising less than 1% of births. The age, sex, and serostatus of all nine elephants are summarized in Table 1. Seropositive elephants were defined by the presence of EEHV1A-specific antibodies against any of the EEHV1A ORFQ proteins belonging to three major clades, as determined by the Luciferase Immunoprecipitation System (LIPS) assay (12, 19). Seronegative elephants had no detectable antibodies against these proteins, with the exception that elephants 5, 8, and 9 were at the cutoff for reactivity against ORFQ clade A at day 0 only (Fig. S1 to S3). Serostatus was further confirmed by assessing reactivity to the EEHV1A glycoprotein trimer complex (gH/gL/gO) as described in the results.

**TABLE 1:** Elephant EEHV1A vaccine cohort

Group	Elephant	Age (yr)	Sex^*a*^	Serostatus^*b*^
Group 1	1	41	F	+
	2	3	F	+
	3	3	M	+
Group 2	4	2	M	−
	5	7	M	−
	6	7	M	−
Group 3	7	2	M	−
	8	2	M	−
	9	1	F	−

^*a*^ M, male; F, female.

^*b*^ Positive (+) or negative (−) serostatus was determined by LIPS or ELISA as described in Materials and Methods.

### Vaccination and serum collection

**Fig 1:**
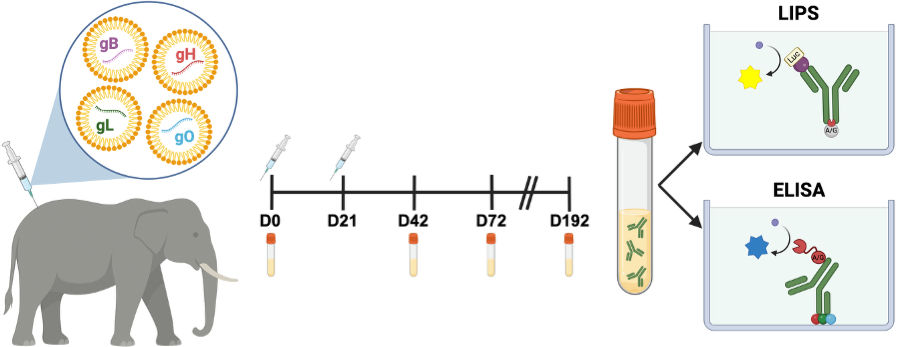
Experimental design of EEHV1A vaccination study. Elephants received two doses of the multiantigen mRNA vaccine (at day 0 and day 21). Sera were collected prior to vaccination and 3 weeks later. A booster dose was administered on day 42, followed by serum collection every 30 days. The luciferase immunoprecipitation system (LIPS) was used to detect antibody responses to individual antigens, whereas the indirect ELISA measured changes in antibody levels to the gH/gL/gO trimer protein. This figure was created using BioRender.com.

The EEHV1A mRNA vaccine targeting glycoproteins gB, gH, gL, and gO was prepared as previously described (17). Briefly, mRNAs encoding each glycoprotein from the EEHV1A Kimba strain (20), including a C-terminal Flag (gB and gL) or HA (gH and gO) epitope tag, were synthesized by the Houston Methodist Center for RNA Therapeutics (Houston, TX) and individually encapsulated in lipid nanoparticles (LNPs) under sterile conditions, followed by filtration through a 0.22 µm filter. Equal amounts (100 µg each) of the four LNP-formulated mRNAs were combined to create a 400 µg total dose in approximately 2 mL of phosphate buffer with 8% sucrose. All vaccines were frozen and stored at −80°C until use, and all vaccines administered in this study were from the same production batch. Elephants in groups 1 and 2 received two intramuscular injections of the 400 µg vaccine, administered 3 weeks apart in either the front or hind leg (Fig. 1). Since no response was observed in group 3 elephants after the initial two vaccinations, two additional vaccines were administered at 3-week intervals, with Day 0 indicating the time point for vaccine #3. Serum samples were collected on day 0 (pre-vaccination) and day 42 (3 weeks post-booster) and then monthly up to day 192. Blood was drawn via venipuncture of the ear veins into red-top tubes. After clotting, serum was aliquoted and stored at –20°C.

### LIPS assay

LIPS assays were performed as previously described (5, 6, 12). Briefly, codon-optimized cDNAs encoding EEHV1A gB, gH, gL, and gO were cloned into the pGAUS3 vector to generate *Gaussia* luciferase fusion proteins. Expression plasmids with HA- and Flag-tagged *Gaussia* luciferase were also constructed; 1 × 10^6^ 293 T cells/well of a six-well tissue culture dish were transfected with expression plasmids in a total of 2.0 mL media, and after 48 h, either supernatants or cell extracts were harvested for luminescence determination on a Glomax 20/20 luminometer (Promega). *Gaussia* luciferase does contain a nuclear export signal, but not all fusion proteins get secreted. gB, gO, and Flag or HA epitope-tagged *Gaussia* luciferase fusion proteins are expressed abundantly in the supernatant of transfected cells as determined by luminescence units (LU), while gH and gL *Gaussia* luciferase fusion proteins are largely cell-associated, and cell extracts are used for these biomarkers. Cell supernatants were prepared by a short spin to remove floating cells and debris, and 1 µl was used to measure *Gaussia* luciferase activity, which often exceeds 1 × 10^8^ LUs/µL. For nonsecreted fusion proteins, supernatants were removed; cells were washed 2× with PBS and lysed with *Renilla* luciferase assay lysis buffer (Promega); and 1 µL was tested to measure *Gaussia* luciferase activity. Fusion proteins are frozen down into aliquots and stored at −80℃. Each transfection yields enough fusion protein for hundreds to thousands of assays. All assays for this study were conducted using the same lots of each fusion protein, respectively. Linear ranges were determined for each fusion protein by serially diluting a seropositive serum sample from 1:10 to 1:56,250 (Fig. S4A through G). To assess antibody levels within the linear range of each assay, sera were diluted 1:500 for gB and gH, and all other serum samples were diluted 1:10 and mixed with 1 × 10^7^ LU for each fusion protein. Immune complexes were captured using protein A/G sepharose beads as described previously (5, 6, 12). After extensive washing, luciferase activity was measured by adding *Renilla* substrate buffer, and relative light units (RLUs) were recorded over 5 s using an OmniStar luminometer (BMG Labtech). Reported values represent the average RLUs over this period.

### Recombinant gH/gL/gO expression and purification

Codon-optimized cDNAs for EEHV1A Kimba strain (20) gH (residues 30–706), gL (residues 57–304), and gO (residues 1–212) were cloned into the pCI vector with C-terminal StrepTag, MycTag, or FlagTag, respectively. The expression and purification scheme for the gH/gL/gO trimer is illustrated in Fig. S5. Briefly, human embryonic kidney 293 (293F) cells were co-transfected with gH (58 µg), gL (24.25 µg), and gO (17.75 µg) plasmids. Enhancers from the Expi293 Expression System (Gibco) were added 24 h post-transfection, and supernatants were harvested after an additional 72 h. Cell debris was removed by centrifugation and filtration through a 0.2 µm PES membrane. The trimer was purified using StrepTrap XT affinity chromatography followed by size-exclusion chromatography (SEC) on Superdex 200 columns (Cytiva) (Fig. S6A). Protein quality was assessed by sodium dodecyl sulfate-polyacrylamide gel (SDS-PAGE), Coomassie staining, and immunoblotting under both denaturing and non-denaturing conditions (Fig. S6B through D). Additionally, gH/gL/gO protein complexes were further characterized by differential scanning fluorimetry (DSF), redshift microfluidic modulation spectroscopy (MMS), and size-exclusion chromatography coupled with multi-angle light scattering (SEC-MALS).

### DSF

Extrinsic differential scanning fluorimetry (DSF) was performed on a LightCycler 480 (Roche); 19 µL of 0.5 mg/mL gH/gL/gO protein was mixed with 1 µL 500× Sypro Orange and run in triplicate. BSA at 2 mg/mL was used as a control. Fluorescence was measured twice per second from 29°C to 90°C. Data are shown as the first derivative of the melt curves (Fig. S7).

### MMS

As a further examination of protein quality, secondary structures of purified gH/gL/gO protein were assessed via microfluidic modulation spectroscopy (MMS) on a fully automated Aurora TX RedShift Biosystem (RedShift Bio). Proteins were dialyzed overnight prior to exchange into 25 mM HEPES, 150 mM NaCl, pH 7.5 at 0.5 mg/mL on a Zebra spin desalting column (Thermo Fisher Scientific) to ensure buffer matching. Samples were introduced into a high-precision microfluidic flow cell with 7–30 psi backing pressure and 1 Hz modulation. Absorbance was recorded using a cadmium detector following excitation by a mid-infrared quantum cascade laser. Spectra were collected across the amide I region (1,700–1,600 cm^−1^). Differential absorbance spectra were obtained by subtracting the buffer signal from the sample signal and averaging the resulting values. This similarity plot is shown in Fig. S8A.

Differential absorbance spectra were processed using *delta* analytics software (21), with a nominal displacement factor of 0.6 and fit to a lysozyme model across 1,688–1,620 cm^−1^. Protein concentration fit and displacement factors were 30% and 20%, respectively. Second-derivative spectra were smoothed using Savitzky-Golay filtering (22), with a 19-wavenumber window, baselined at both ends, inverted, and fit to Gaussian functions. Gaussian peak areas were used to calculate the percentage contributions of higher-order structural (HOS) elements (Fig. S8B).

### SEC-MALS

Size-exclusion chromatography coupled with multi-angle light scattering (SEC-MALS) was run on a Superose 6 column (Cytiva) connected to a Dawn MALS instrument (Wyatt Technology) equipped with a 661 nm laser. This instrument is linked to an HPLC system (Agilent), with UV absorbance detection at 280 nm and an Optilab (Wyatt Technology) for differential refractive index (dRI) measurements. Approximately 0.1 mg of gH/gL/gO at 0.9 mg/mL was injected. Data analysis was performed using Astra software v 8.2.2 (Wyatt Technology). Calibration was performed with 2 mg/mL BSA with PBS as the running buffer at a flow rate of 0.5 mL/min. Molecular weight distribution is shown in Fig. S9.

### EEHV1A ELISA

For ELISA, Immulon 2HB 96-well plates (Thermo) were coated overnight at 4°C with 30 ng/well of gH/gL/gO trimer in carbonate coating buffer (pH 9.6). All reagents were added in volumes of 100 µL/well and incubated for 1 h at 37 °C unless otherwise specified. Plates were washed three times with 200 µL of imidazole-buffered saline containing Tween 20 (SeraCare) and blocked with 5% milk in wash buffer. After washing, elephant serum diluted 1:100 in 5% milk was added. Plates were then washed and incubated with 0.5 µg/mL horseradish peroxidase (HRP)-conjugated recombinant Protein A/G (Thermo). Following a final wash, 3,3′,5,5′-tetramethylbenzidine (TMB) substrate (SeraCare) was added and incubated in the dark for 18 min at room temperature. The reaction was stopped with 0.32 M sulfuric acid, and absorbance was measured at 450 nm using a Synergy H1 microplate reader (Agilent BioTek). The linear range for this assay was determined by diluting positive serum from three seropositive elephants (Fig. S4H). ΔOD values were calculated by subtracting background absorbance (serum in uncoated wells) from antigen-specific signals.

### Statistical analysis

Statistical analyses were performed using GraphPad Prism 10. LIPS data were grouped by serostatus and vaccination schedule. For normally distributed data, a two-way or one-way ANOVA with Dunnett’s multiple comparisons test was applied for cohort or individual data, respectively. A *P*-value ≤ 0.05 was considered statistically significant. Cutoff values for sensitivity and specificity of each viral antigen were determined as the mean antibody titer of no-serum control samples plus five standard deviations. The cutoff for gH/gL/gO trimer ELISA was determined by receiver operating characteristic (ROC) analysis using serum from eight EEHV1A seropositive elephants, six EEHV1A seronegative elephants, and four seronegative elephants before and after vaccination.

## RESULTS

### Group 1: seropositive elephants

The first three elephants (Table 1, group 1) to receive the multiantigen mRNA vaccine were known to be seropositive for at least one of three major clades of EEHV1A ORFQ proteins (Fig. S1 to S3), which are only expressed by EEHV1 strains and thus were presumed to be chronically infected with EEHV1A. No adverse side effects, including anaphylaxis, were observed following the initial vaccination. However, elephant 3 developed mild swelling at the injection site after the booster dose, which self-resolved within 4 days. Throughout the study period, none of the elephants exhibited changes in blood cell counts or serum chemistries outside their normal ranges.

**Fig 2:**
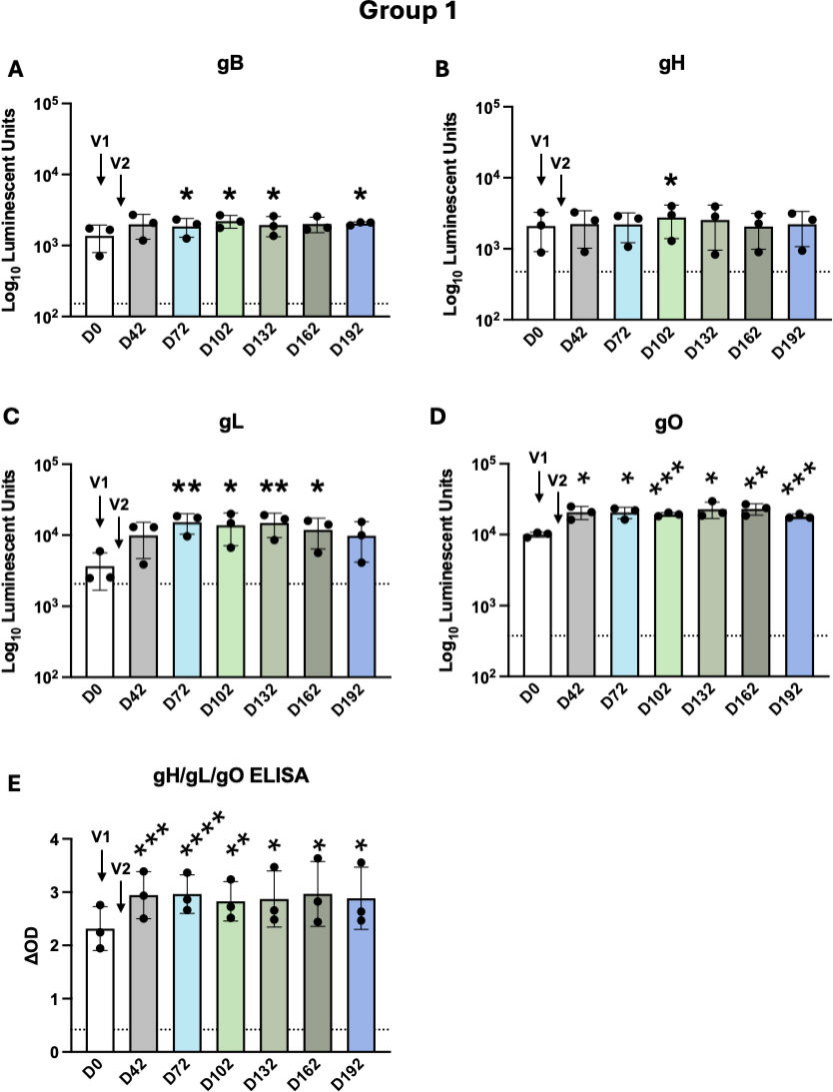
Antibody response to EEHV1A multi-antigen mRNA vaccine in seropositive elephants (Group 1). Detection of anti-gB (**A**), -gH (**B**), -gL (**C**), and -gO (**D**) antibodies by LIPS assay was assessed prior to vaccination through 192 days post-vaccination. The results are shown as luminescent units plotted on a log_10_ scale. Each point represents the average of two technical replicates for a single elephant and is representative of at least two independent experiments. The dotted line represents the limit of detection determined by the mean of the no serum negative controls plus five standard deviations. Antibodies against gH, gL, and gO were collectively assessed by indirect ELISA, where each data point represents the average of two technical replicates from independent experiments for each elephant (**E**). The results are depicted as ΔOD (signal from uncoated well subtracted from signal in a coated well). The cutoff was determined by the ROC curve. Statistical significance was determined by two-way repeated measures ANOVA with Dunnett’s test. V1 and V2 represent the approximate timing of prime (D0) and boost (D21) vaccine doses, respectively. *P < 0.05, **P < 0.01, ***P < 0.001, **** *P* < 0.0001.

We used LIPS assays to evaluate antibody responses to each individual EEHV antigen encoded by the vaccine. Antibody levels for gB increased modestly following vaccination with some marginal increase for gH (day 102), although baseline levels for these antigens were relatively high (Fig. 2A and B). In contrast, antibody levels against gL and gO increased more substantially between days 42–72 (Tables 2 and 3) post-vaccination and remained elevated through day 192 (Fig. 2C and D; Tables 2 and 3). Antibody levels against the gH/gL/gO trimer, measured by ELISA, increased consistently in all three elephants following vaccination (Fig. 2E; Tables 2 and 3). Individual antigen analysis indicates that the trimer reactivity was largely driven by responses to gL and gO in the seropositive group. Vaccine response profiles for each individual elephant are shown in Fig. S10. These findings suggest that in EEHV1A-experienced, seropositive elephants, the vaccine was well tolerated and elicited sustained antibody responses for up to 6 months, particularly targeting the gH/gL/gO trimeric complex.

**TABLE 2:** Relative change in antibodies after vaccination in seropositive and seronegative elephants^*c*^

Antigen	gB		gH		gL		gO		gH/gL/gO	
Serostatus^*d*^	+	−	+	−	+	−	+	−	+	−
D0 mean	1,370	313	9,030	251	3,660	1,504	10,016	143	2.32	0.18
D42 mean	1,989	2,788	10,605	893	9,939	14,354	20,557	622	2.94	1.00
% change from D0 to D42	45.12	790.70	17.44	255.06^*a*^	171.58	854.13^*a*^	105.24	335.24	27.06^*b*^	461.56^*a*^

^*a*^ *P* < 0.05.

^*b*^ *P* < 0.01.

^*c*^ LIPS and ELISA results were collectively analyzed based on serostatus prior to vaccination. Numbers are shown as the average luminescent units (gB, gH, gL, and gO) or ΔOD (gH/gL/gO). Statistical significance was determined by a paired-samples *t*-test.

^*d*^ Positive (+) or negative (−) serostatus was determined by LIPS or ELISA as described in Materials and Methods.

**TABLE 3:** Relative change in antibodies 72 days after vaccination in seropositive and seronegative elephants^*c*^

Antigen	gB		gH		gL		gO		gH/gL/gO	
Serostatus^*d*^	+	−	+	−	+	−	+	−	+	−
D0 mean	1,370	313	2,094	251	3,660	1,504	10,016	143	2.32	0.18
D72 mean	1,861	2,762	2,211	811	15,199	8,799	20,445	479	2.96	0.94
% change from D0 to D72	35.82	782.40	5.61	222.44	315.33^*a*^	484.92	104.12^*a*^	235.13	27.93^*b*^	429.49^*a*^

^*a*^ *P* < 0.05.

^*b*^ *P* < 0.01.

^*c*^ LIPS and ELISA results were collectively analyzed based on serostatus prior to vaccination. Numbers are shown as the average luminescent units (gB, gH, gL, and gO) or ΔOD (gH/gL/gO). Statistical significance was determined by a paired-samples *t*-test.

^*d*^ Positive (+) or negative (−) serostatus was determined by LIPS or ELISA as described in Materials and Methods.

### Group 2: EEHV1A seronegative elephants

**Fig 3:**
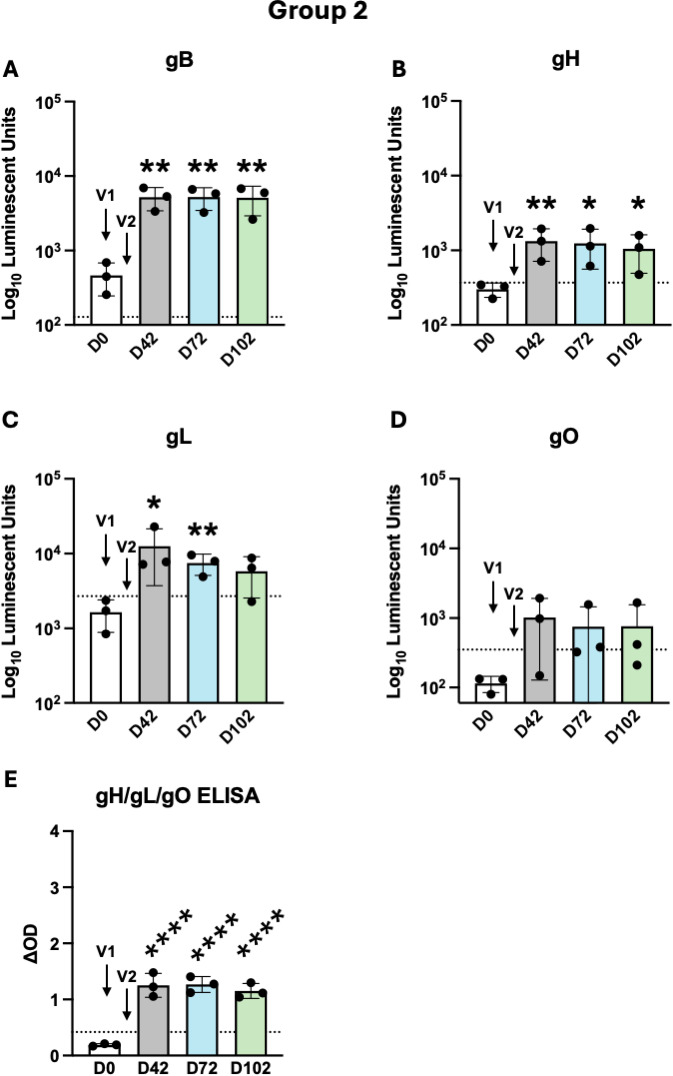
Antibody response to EEHV1A multi-antigen mRNA vaccine in seronegative elephants (Group 2). Detection of anti-gB (**A**), -gH (**B**), -gL (**C**), and -gO (**D**) antibodies by LIPS assay was assessed prior to vaccination through 102 days post-vaccination. Each point represents the average of two technical replicates for a single elephant and is representative of at least two independent experiments. The dotted line represents the limit of detection determined by the mean of no serum negative controls plus five standard deviations. Antibodies against gH, gL, and gO were collectively assessed by indirect ELISA (**E**). Each data point represents the average of two technical replicates from two independent experiments for each elephant. The cutoff was determined by ROC curve. Statistical significance was determined by two-way repeated measures ANOVA with Dunnett’s test. V1 and V2 represent the approximate timing of prime (D0) and boost (D21) vaccine doses, respectively. *P < 0.05, **P < 0.01, ****P < 0.0001.

Elephants in Group 2 were seronegative for EEHV1A ORFQ but likely had prior exposure to EEHV4 and/or EEHV5, as indicated by high baseline reactivity to gB, which is relatively conserved among the different EEHV types (Fig. 3A) and is consistent with previous studies (12, 19). Elephants 5 and 6 received preemptive plasma transfusions from EEHV seropositive donors in May and June of 2024, which resulted in a transient increase in detectable antibodies against ORFQ and the gH/gL/gO trimer that declined to seronegative levels prior to receiving the mRNA vaccine in October and November of 2024 (Fig. 3B through E) (18).

Increases in EEHV1A gB antibody levels were observed through day 102 (Fig. 3A). Prior to vaccination, all three elephants had antibody levels below the detection threshold for gH, gL, and gO (Fig. 3B through D). Post-vaccination, titers against all three antigens rose above the cutoff and remained elevated through day 102. Significant reactivity against the trimer was observed through day 102, although titers began to decline from their peak (Fig. 3E). The trimer reactivity likely reflects cumulative responses to gH, gL, and gO, as supported by the LIPS data. Vaccine response profiles for each individual elephant are shown in Fig. S11. While post-vaccination trimer antibody levels were substantially higher than baseline, they approached approximately 50% of those observed in Group 1 elephants prior to their vaccination.

### Group 3: additional seronegative elephants

**Fig 4:**
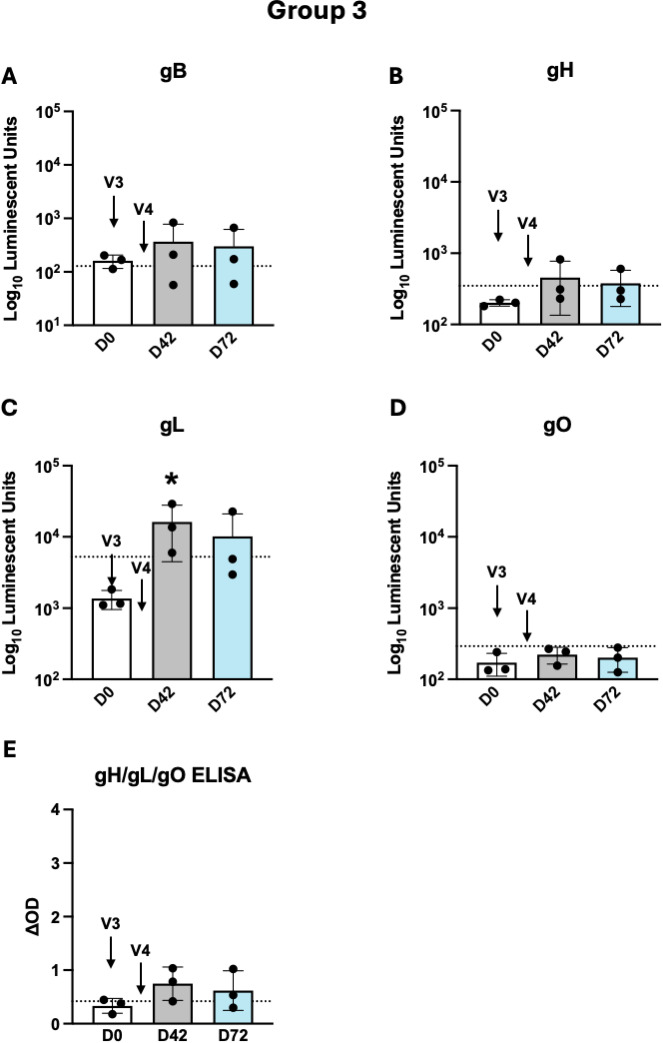
Antibody response to EEHV1A multi-antigen mRNA vaccine in seronegative elephants (Group 3). Detection of anti-gB (**A**), -gH (**B**), -gL (**C**), and -gO (**D**) antibodies by LIPS assay was assessed prior to vaccinations 3 and 4 through 72 days post-vaccination. Each point represents the average of two technical replicates and is representative of at least two independent experiments. The dotted line represents the limit of detection determined by the mean of no serum negative controls plus five standard deviations. Antibodies against gH, gL, and gO were collectively assessed by indirect ELISA (**E**). Each data point represents the average of two technical replicates from two independent experiments for each elephant. The cutoff was determined by ROC curve. Statistical significance was determined by two-way repeated measures ANOVA with Dunnett’s test. *P < 0.05.

Group 3 elephants generated little to no detectable changes in antibody levels after their first two vaccinations. Since this outcome was unique to this group, two additional doses were administered to these elephants. Data shown for Group 3 follows the third and fourth vaccinations. This group exhibited a somewhat different response pattern compared to Group 2. Post-vaccination gB titers trended slightly lower, but reactivity toward gL was significantly higher post-vaccination, decreasing slightly at day 72 (Fig. 4A and C). A slight increase in titers against gO was also observed, although it did not reach statistical significance (Fig. 4D). Trimer reactivity mirrored the patterns observed for individual antigens, although peak titers were lower than those of Group 2 elephants (Fig. 4E). Vaccine response profiles for each individual elephant are shown in Fig. S12.

### Specificity and immunogenicity of EEHV mRNA vaccine

Serostatus of each elephant was confirmed prior to vaccination by detection of EEHV1A ORFQ proteins via LIPS assay (Fig. S1 to S3). Lack of reactivity to ORFQ by seronegative elephants over the course of the study implies that they did not develop a primary infection with EEHV1A. This suggests that any increases in antibody titers against glycoproteins encoded in the vaccine are a direct result of vaccination rather than a response to EEHV infection. Interestingly, no reactivity against HA or Flag epitope tags present on the vaccine-expressed glycoproteins was detected, as assessed by immunoreactivity against HA- or Flag-tagged Gaussia Luciferase proteins in LIPS assays (Fig. S13), suggesting that all observed responses were against the EEHV glycoproteins and not the epitope tags. The overall effectiveness of the vaccine for generating antibody responses, particularly in the seronegative groups, is summarized in Tables 2 and 3.

## DISCUSSION

In this study, we evaluated the immunogenicity of a novel multiantigen mRNA vaccine targeting EEHV1A in Asian elephants. Vaccination of both EEHV1A-naïve and chronically infected elephants elicited increases in antibody titers against components of the conserved viral attachment complex—specifically glycoproteins gH, gL, and gO—with responses maintained for up to 6 months post-vaccination in some elephants. EEHV1A-seronegative elephants mounted variable responses against gB. Antibody titers generally peaked at day 42 (21 days following the booster dose) and, although they declined over time, are sustained above baseline up to day 192, depending on the antigen. These findings demonstrate that Asian elephants, particularly those naïve to EEHV1A, can mount specific immune responses to an mRNA-based vaccine.

### Variable responses observed in seronegative elephants

As previously noted, two additional vaccine doses were required to elicit detectable immune responses in Group 3 elephants. Given the consistently high standards of care in Association of Zoos and Aquariums (AZA)-accredited facilities, including behavioral management, nutrition, veterinary care, and environmental conditions, extrinsic factors are unlikely to account for this delayed response (23, 24). All vaccine doses were from the same production batch, suggesting that batch effects were unlikely to contribute to this effect. More plausible explanations include: (i) the younger average age of elephants in Group 3 compared to Groups 1 and 2, suggesting a potential age-related effect on immune responsiveness, and/or (ii) the possibility that Group 3 elephants are inherently low responders, either in an antigen-specific manner or more broadly across vaccines, potentially reflecting individual genetic variation (23–25). Notably, one of the elephants in group 3 was rejected by its dam and was bottle-fed for the first 2 years and has consistently exhibited lower than average total immunoglobulin levels. The other two calves are twins, an extremely rare occurrence in elephants comprising less than 1% of births, which often are stillborn or incur high mortality rates. Whether immune system development and maturation may be compromised in elephant twins due to factors such as *in utero* nutritional competition, reduced maternal antibody transfer through shared colostrum resources, or developmental delays associated with lower birth weights remains unstudied. These three individuals with documented early life challenges may represent a subpopulation with inherently different immunological baselines, potentially explaining their attenuated vaccine responses compared to elephants with typical early development. Elephants with similar characteristics may require a more extensive and aggressive vaccination schedule in order to induce effective anti-EEHV1A immunity. Continued vaccination and monitoring of a larger cohort will help determine the prevalence of low responsiveness and whether such responses still confer protection against EEHV-HD.

### Is the vaccine response sufficient to protect against EEHV hemorrhagic disease?

Elephants are known to transfer substantial levels of antibodies transplacentally to their calves (12, 26), and these antibodies can persist for up to 2 years or longer. Given that the average half-life of mammalian antibodies is approximately 2–4 weeks, maternal antibodies would typically be depleted within 9–12 months. This discrepancy suggests that calves may receive ongoing antibody supplementation through maternal milk. Most EEHV-HD cases occur in elephants older than 2 years, especially those with low to undetectable antibodies, suggesting that high levels of anti-EEHV antibodies may be protective against severe disease following primary infection. Prior infection with other EEHV types (i.e., EEHV1B, 4, and/or 5) does not appear to generate cross-protective immunity as several calves have died from EEHV-HD associated with EEHV1A, and evidence of previous infection with at least one of the other EEHV types (12, 13, 27, 28).

To date, EEHV has not been successfully cultivated *in vitro*, and no pseudotyped virus system exists to investigate viral attachment processes or to establish neutralization assays. Without these tools, the specific antibody targets and protective thresholds remain unknown. However, we hypothesize that antibodies against gB and the gH/gL/gO trimer complex, components of the conserved herpesvirus attachment and fusion machinery, may be critical. While passive antibody transfer can be protective for some herpesvirus infections, cellular immunity is generally essential for viral control and clearance (29–40). Deficiencies in T cell responses often correlate with severe disease outcomes.

Preclinical studies in mice using the same multiantigen mRNA vaccine demonstrated both humoral and Th1-type bifunctional T-cell responses (17). Although cohort-wide T cell analysis in elephants was not feasible in this study due to logistical limitations in acquiring and processing viable peripheral blood mononuclear cells (PBMCs) on site, we anticipate that similar cellular responses were likely induced. Thus, while vaccine-induced antibody responses in young elephants may be narrower and lower than maternal antibody levels, primed T cell responses may provide complementary protection against primary EEHV1A infection.

### Antibody responses against glycoprotein O

In contrast to humans, glycoprotein O appears to be significantly more immunogenic in elephants than CMV gO is in humans. Human cytomegalovirus gO is substantially larger than the EEHV1A version (376 versus 181 amino acid residues) and correspondingly contains more potential O-linked (55 vs. 26) and N-linked glycosylation sites (11 vs. 7). Consistent with heavy glycosylation, EEHV1A gO present in EEHV1A trimer complexes (Fig. S6D) migrates at a significantly higher molecular weight than predicted from the protein sequence alone. While extensive glycosylation may shield CMV gO from immune recognition in humans, structural differences in EEHV gO presentation within the trimer complex or distinct immune recognition patterns in elephants could explain these differential antibody responses. Structural studies of the EEHV1A trimer complex combined with further elucidation of MHC and antibody recognition domains in elephants may help resolve these issues.

### Strengths and limitations of LIPS versus ELISA

As previously reported, the LIPS assay requires only small serum volumes, which is advantageous when sampling from calves. LIPS also detects immunoreactivity in the liquid phase, potentially preserving native protein conformations. We have also developed an ELISA using the predicted gH/gL/gO trimer complex, commonly observed in Betaherpesviruses. While also requiring a small volume of serum, this format may allow the detection of conformational epitopes formed by the assembled complex. However, a limitation of the ELISA is the inability to discern how the contributions of gH, gL, and gO proteins individually drive the observed reactivity. In contrast, LIPS can provide more granular data on EEHV antigen-specific responses, offering complementary insights into immune recognition.

### Will booster vaccines be required?

Our data show that antibody titers peak between days 42 and 72, following the initial vaccine dose. In EEHV1A-naive elephants, titers against the trimer complex started declining between days 72 and 102 but remained above baseline. This pattern underlies the importance of continued screening and suggests that booster doses may be necessary until natural infection occurs.

Several vaccine platforms require a series of 3–4 immunizations to establish a long-term cellular immune response. However, numerous studies suggest that a heterologous prime-boost strategy, using a protein subunit vaccine as a booster following mRNA priming, may elicit more robust immunity than either platform alone (41–44). We have conducted preliminary studies in mice using adjuvanted protein subunit vaccines against EEHV1A with either gB or gH/gL (15, 16) and envision a potential regimen where subunit boosters with gB and the trimer complex described in this current study are administered after the initial mRNA series or alternated with the mRNA vaccine as needed based on serological testing.

### Timing of vaccination relative to maternal antibody decline

In this study, we targeted elephants with low to undetectable EEHV1A antibody levels. However, as evidence of vaccine efficacy accumulates, it may be advantageous to initiate vaccination in calves as early as 1–2 years of age, even in the presence of maternal antibodies. Although determining serostatus in young elephants can be challenging, vaccination starting at 12 months (or earlier) old could be implemented until serostatus is confirmed or natural infection and lifelong immunity are established.

### Conclusion

In summary, this novel multiantigen mRNA vaccine targeting EEHV1A, the first mRNA vaccine to be used in elephants, elicits a robust immune response in Asian elephants without adverse clinical reactions. This platform represents a significant advancement toward the development of an effective vaccine against EEHV1A and the prevention of EEHV-HD, supporting the Asian elephant population and species conservation.

## Data Availability

The data supporting the findings of this study are available from the corresponding author upon reasonable request. All data generated or analyzed during this study are included in this article.

## SUPPLEMENTAL MATERIAL

Supplemental figures jvi.00388-26-s0001.pdf Fig. S1 to S13.

ASM does not own the copyrights to Supplemental Material that may be linked to, or accessed through, an article. The authors have granted ASM a non-exclusive, world-wide license to publish the Supplemental Material files. Please contact the corresponding author directly for reuse.
