# Supplementary material for: Evaluation of a multiantigen mRNA vaccine against EEHV1A in Asian elephants
Source: J Virol. 2026 Jul 28;100(8):e00388-26. doi: 10.1128/jvi.00388-26 (PMC13483423; doi:10.1128/jvi.00388-26)
Supplement: Supplementary file 1 [file jvi.00388-26-s0001.pdf]

Fig S1

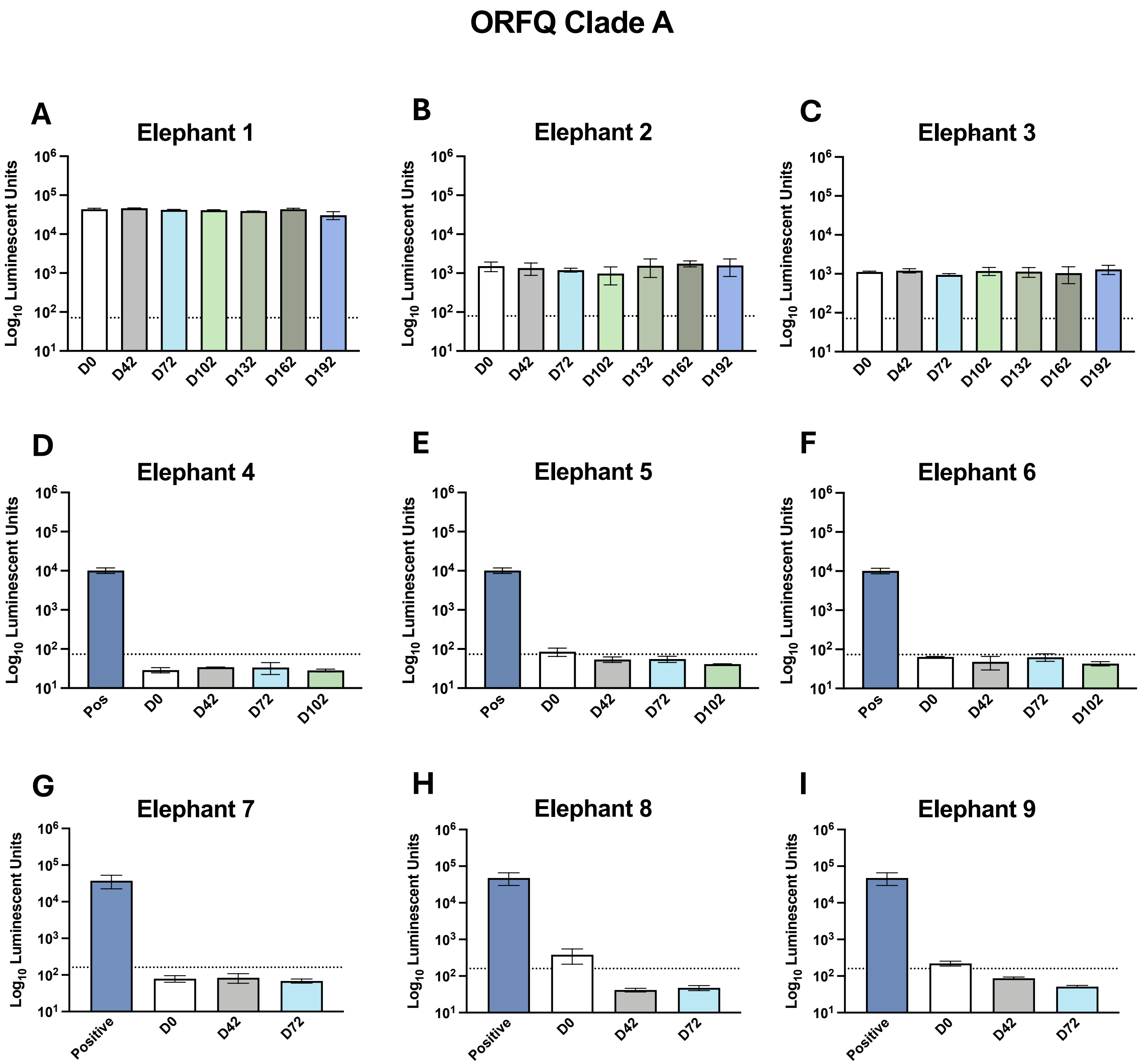

ORFQ Clade A
A
Elephant 1
B
Elephant 2
C
Elephant 3
D
Elephant 4
E
Elephant 5
F
Elephant 6
G
Elephant 7
H
Elephant 8
I
Elephant 9
Log10 Luminescent Units
D0
D42
D72
D102
D132
D162
D192
Pos
Positive

Fig S2

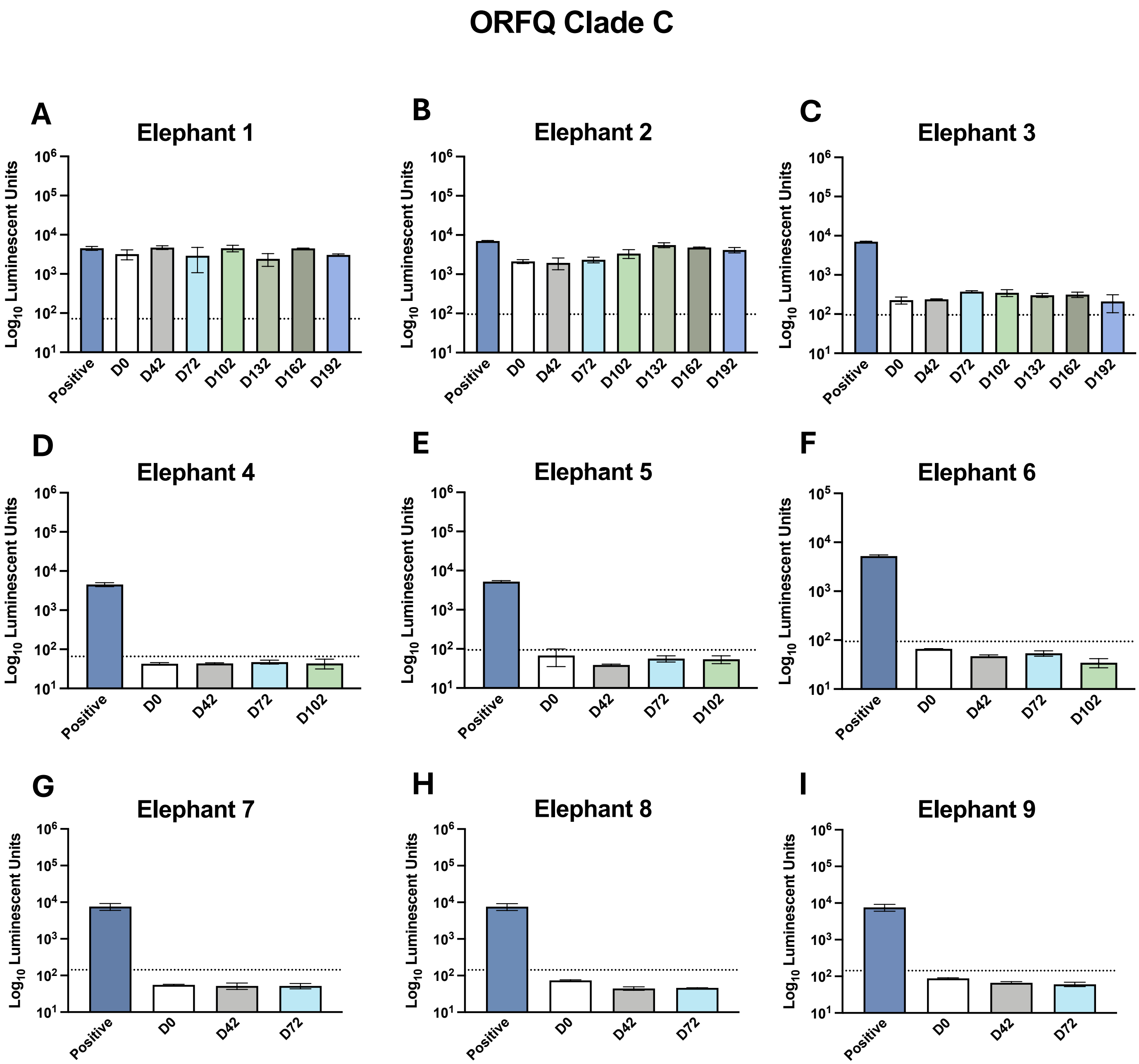
ORFQ Clade C
A
Elephant 1
B
Elephant 2
C
Elephant 3
D
Elephant 4
E
Elephant 5
F
Elephant 6
G
Elephant 7
H
Elephant 8
I
Elephant 9
Log10 Luminescent Units
Positive
D0
D42
D72
D102
D132
D162
D192

Fig S3

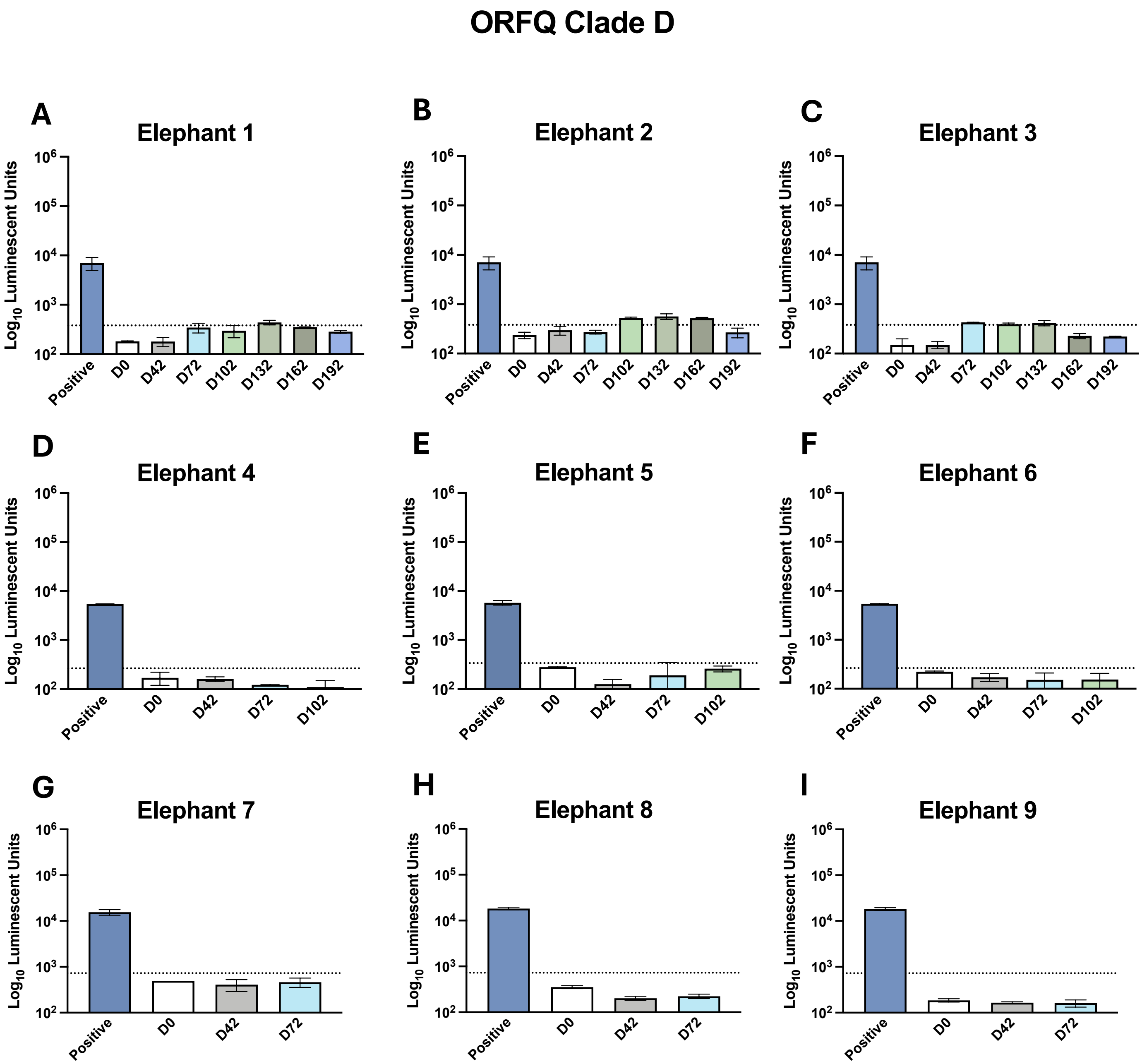

ORFQ Clade D
A
Elephant 1
B
Elephant 2
C
Elephant 3
D
Elephant 4
E
Elephant 5
F
Elephant 6
G
Elephant 7
H
Elephant 8
I
Elephant 9
Log10 Luminescent Units
10^2
10^3
10^4
10^5
10^6
Positive
D0
D42
D72
D102
D132
D162
D192

Fig S4

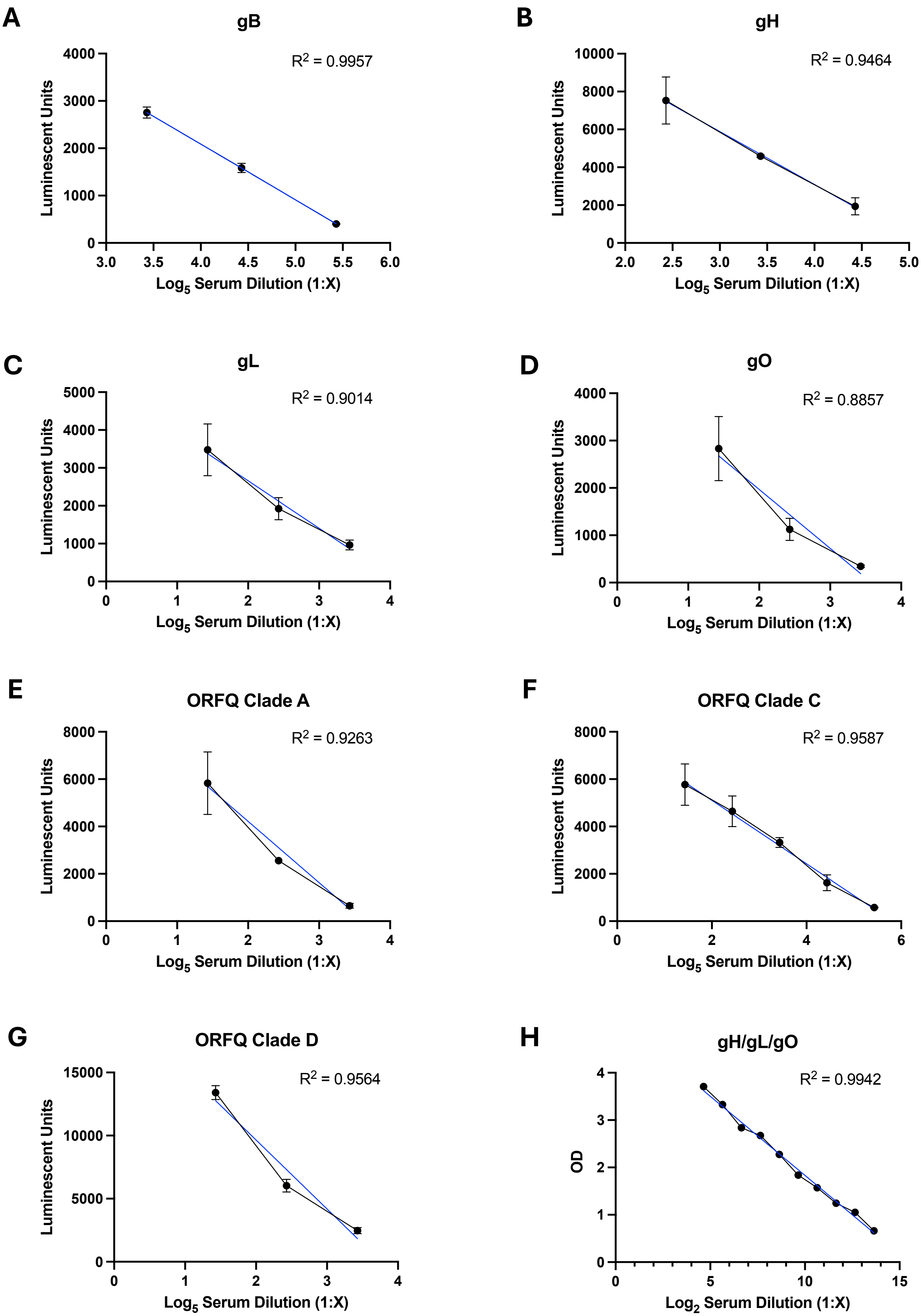

A
gB
R^2 = 0.9957
Luminescent Units
0
1000
2000
3000
4000
3.0
3.5
4.0
4.5
5.0
5.5
6.0
Log_5 Serum Dilution (1:X)
B
gH
R^2 = 0.9464
Luminescent Units
0
2000
4000
6000
8000
10000
2.0
2.5
3.0
3.5
4.0
4.5
5.0
Log_5 Serum Dilution (1:X)
C
gL
R^2 = 0.9014
Luminescent Units
0
1000
2000
3000
4000
5000
0
1
2
3
4
Log_5 Serum Dilution (1:X)
D
gO
R^2 = 0.8857
Luminescent Units
0
1000
2000
3000
4000
0
1
2
3
4
Log_5 Serum Dilution (1:X)
E
ORFQ Clade A
R^2 = 0.9263
Luminescent Units
0
2000
4000
6000
8000
0
1
2
3
4
Log_5 Serum Dilution (1:X)
F
ORFQ Clade C
R^2 = 0.9587
Luminescent Units
0
2000
4000
6000
8000
0
2
4
6
Log_5 Serum Dilution (1:X)
G
ORFQ Clade D
R^2 = 0.9564
Luminescent Units
0
5000
10000
15000
0
1
2
3
4
Log_5 Serum Dilution (1:X)
H
gH/gL/gO
R^2 = 0.9942
OD
0
1
2
3
4
0
5
10
15
Log_2 Serum Dilution (1:X)

Figure S5

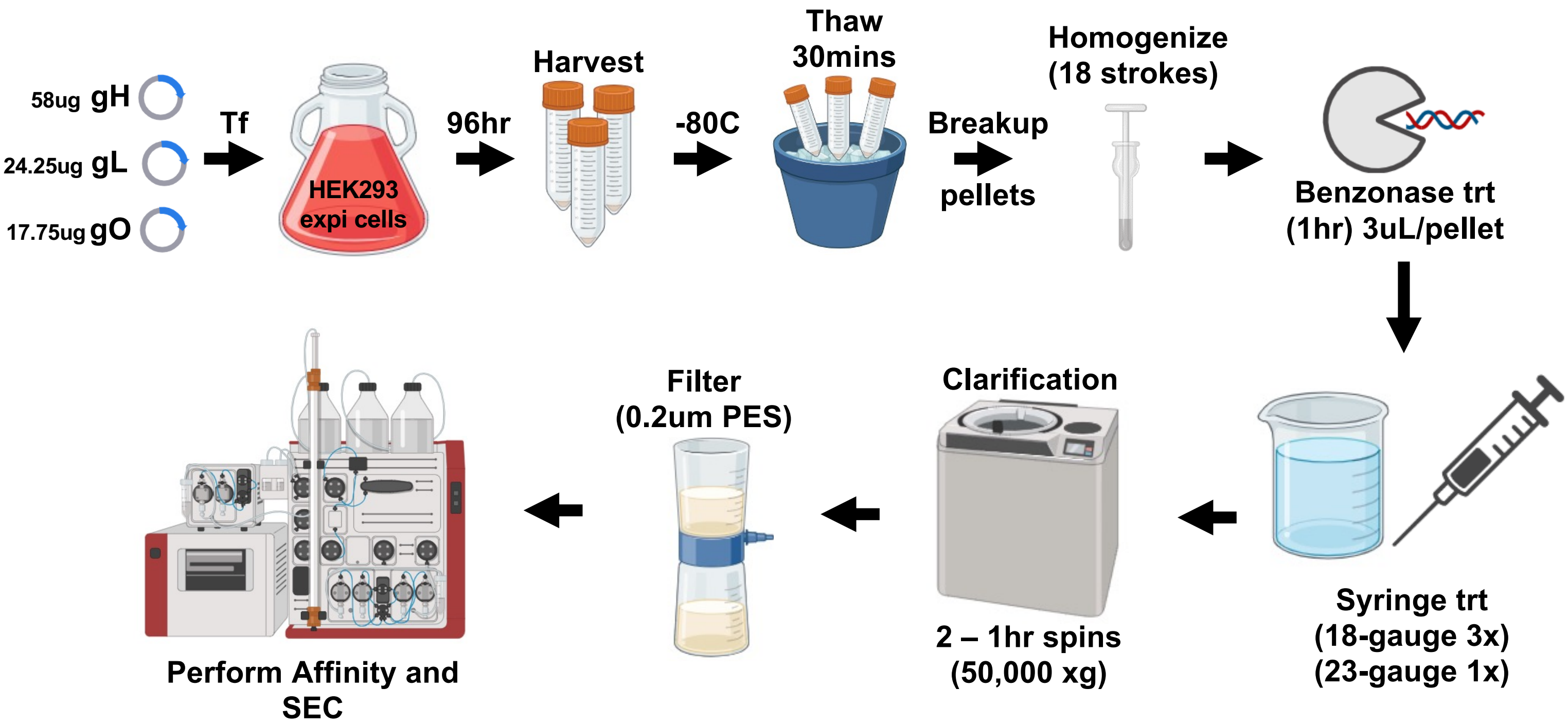

58ug gH
24.25ug gL
17.75ug gO
Tf
HEK293
expi cells
96hr
Harvest
-80C
Thaw
30mins
Breakup
pellets
Homogenize
(18 strokes)
Benzonase trt
(1hr) 3uL/pellet
Syringe trt
(18-gauge 3x)
(23-gauge 1x)
Clarification
2 – 1hr spins
(50,000 xg)
Filter
(0.2um PES)
Perform Affinity and
SEC

Figure S6

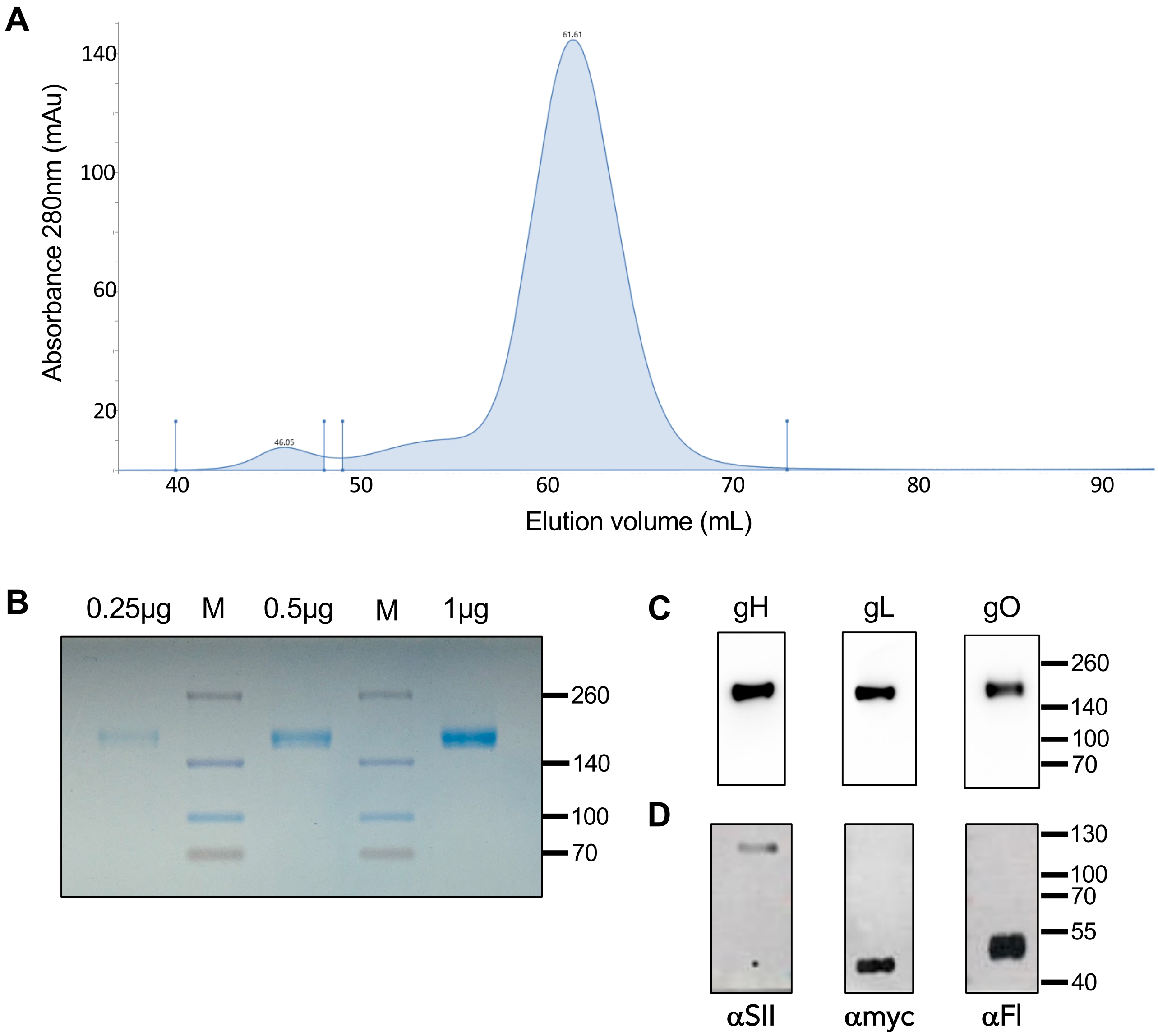
A
Absorbance 280nm (mAu)
140
100
60
20
61.61
46.05
40
50
60
70
80
90
Elution volume (mL)
B
0.25µg
M
0.5µg
M
1µg
260
140
100
70
C
gH
gL
gO
260
140
100
70
D
130
100
70
55
40
αSII
αmyc
αFl

Figure S7

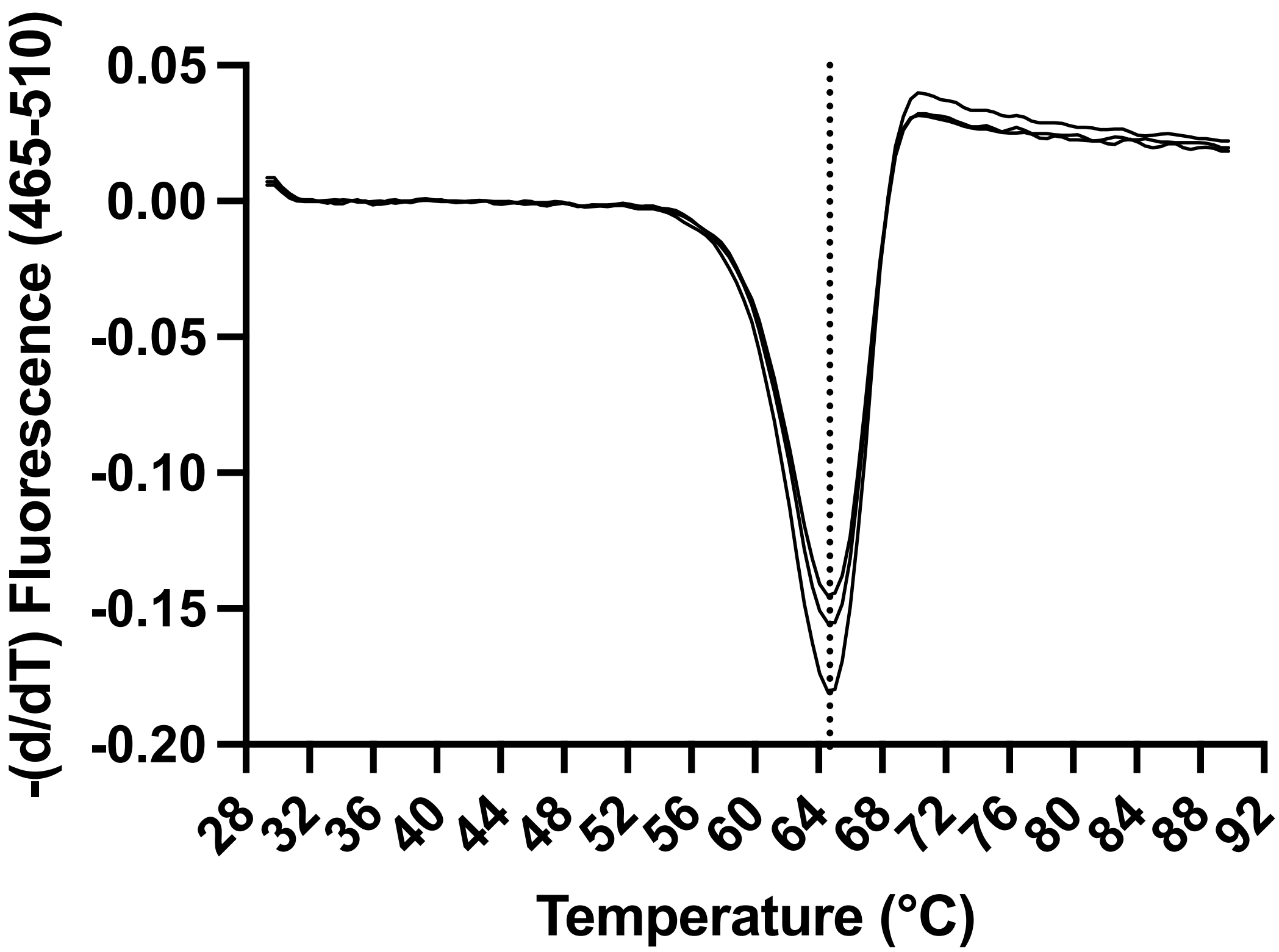

0.05
0.00
-0.05
-0.10
-0.15
-0.20
-(d/dT) Fluorescence (465-510)
28
32
36
40
44
48
52
56
60
64
68
72
76
80
84
88
92
Temperature (°C)

Figure S8

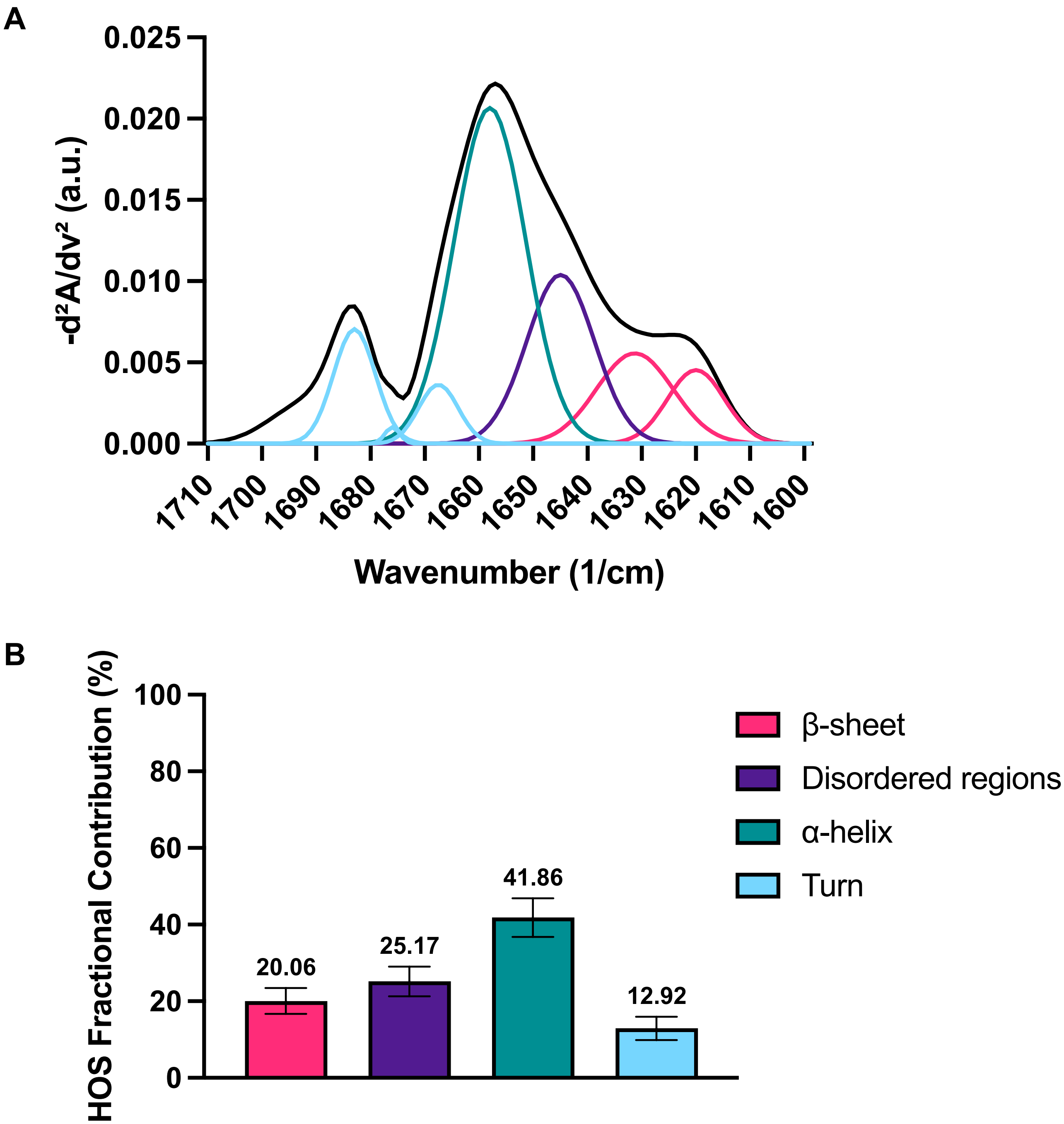
A
0.025
0.020
0.015
0.010
0.005
0.000
-d²A/dv² (a.u.)
1710
1700
1690
1680
1670
1660
1650
1640
1630
1620
1610
1600
Wavenumber (1/cm)
B
HOS Fractional Contribution (%)
100
80
60
40
20
0
20.06
25.17
41.86
12.92
β-sheet
Disordered regions
α-helix
Turn

Figure S9

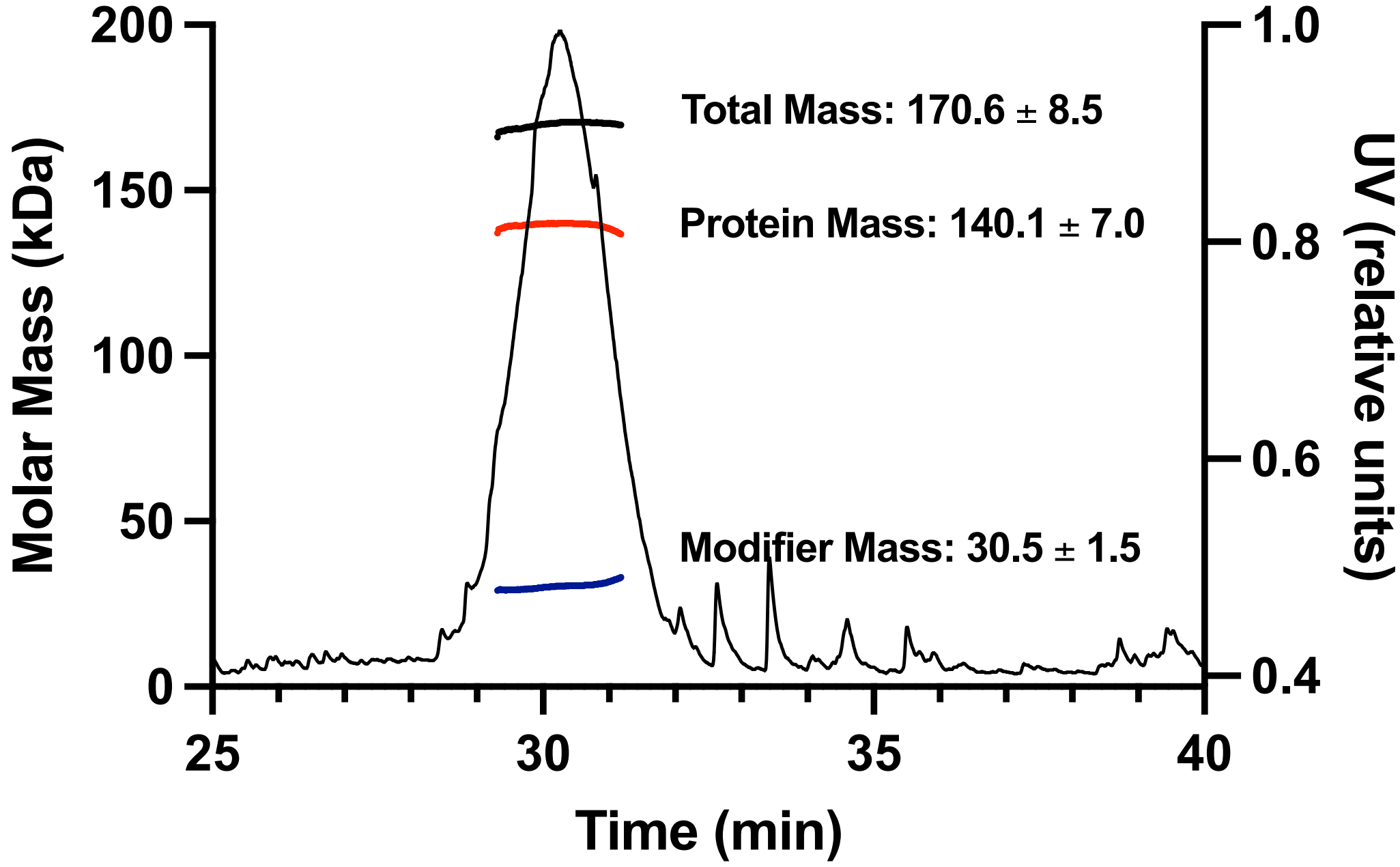

Total Mass: 170.6 ± 8.5
Protein Mass: 140.1 ± 7.0
Modifier Mass: 30.5 ± 1.5
Molar Mass (kDa)
UV (relative units)
Time (min)
200
150
100
50
0
1.0
0.8
0.6
0.4
25
30
35
40

Fig S10

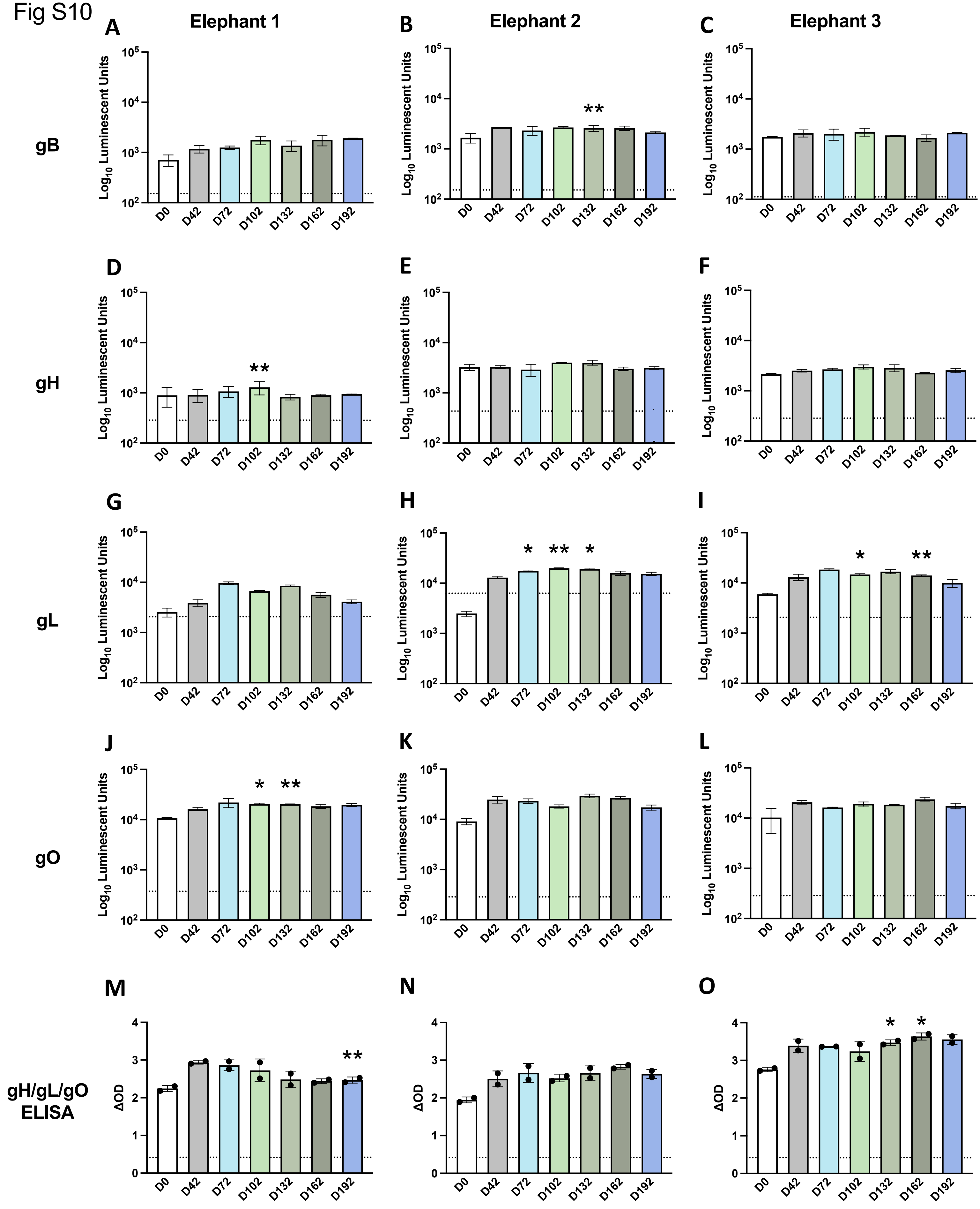
Elephant 1
Elephant 2
Elephant 3
A
B
C
D
E
F
G
H
I
J
K
L
M
N
O
gB
gH
gL
gO
gH/gL/gO ELISA
Log10 Luminescent Units
ΔOD
D0
D42
D72
D102
D132
D162
D192

Fig S11

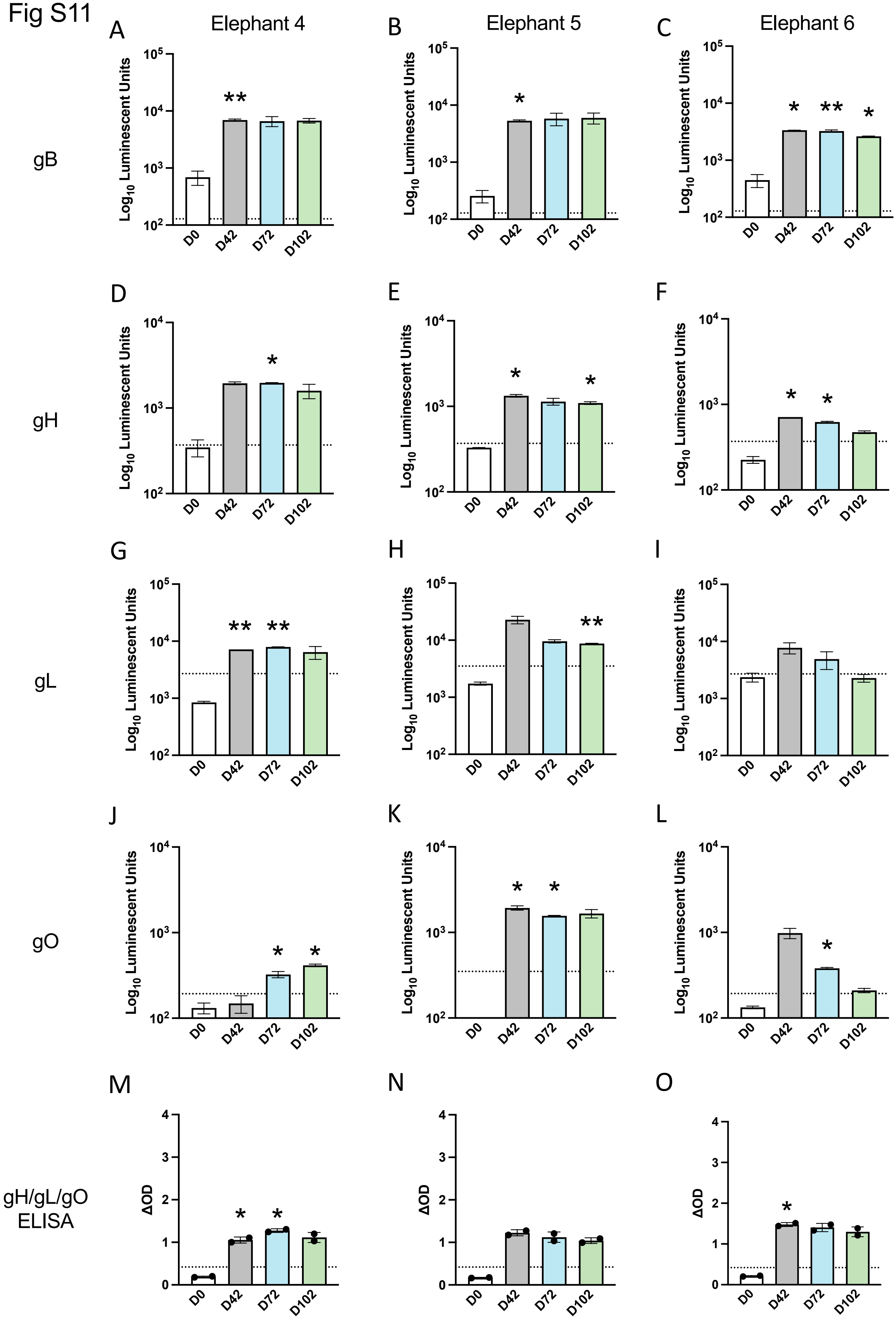

A
Elephant 4
B
Elephant 5
C
Elephant 6
gB
D
E
F
gH
G
H
I
gL
J
K
L
gO
M
N
O
gH/gL/gO ELISA
Log10 Luminescent Units
ΔOD
D0
D42
D72
D102

Fig S12

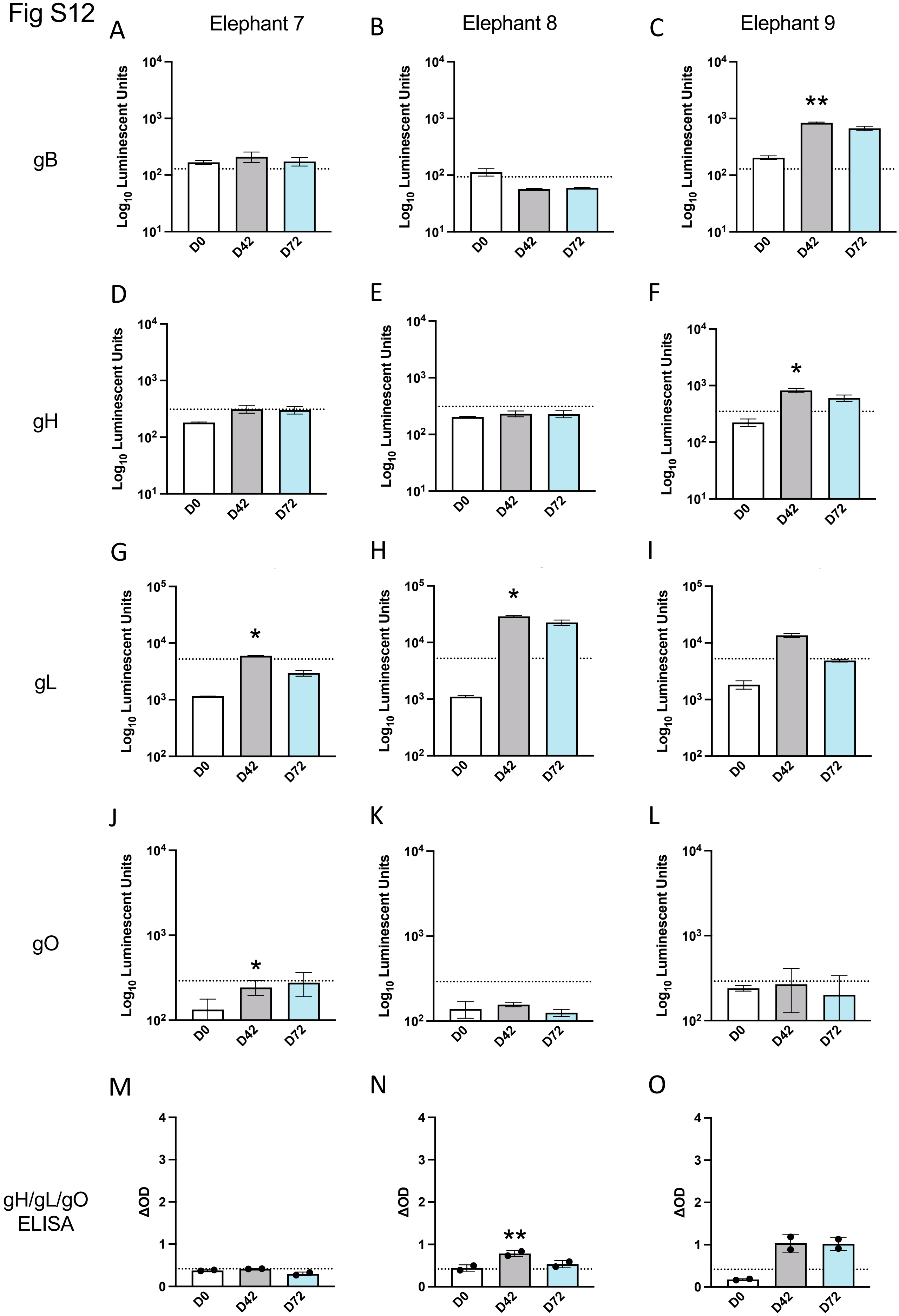

A
B
C
Elephant 7
Elephant 8
Elephant 9
gB
gH
gL
gO
gH/gL/gO ELISA
D
E
F
G
H
I
J
K
L
M
N
O
Log10 Luminescent Units
ΔOD
D0
D42
D72
*
**

Fig S13

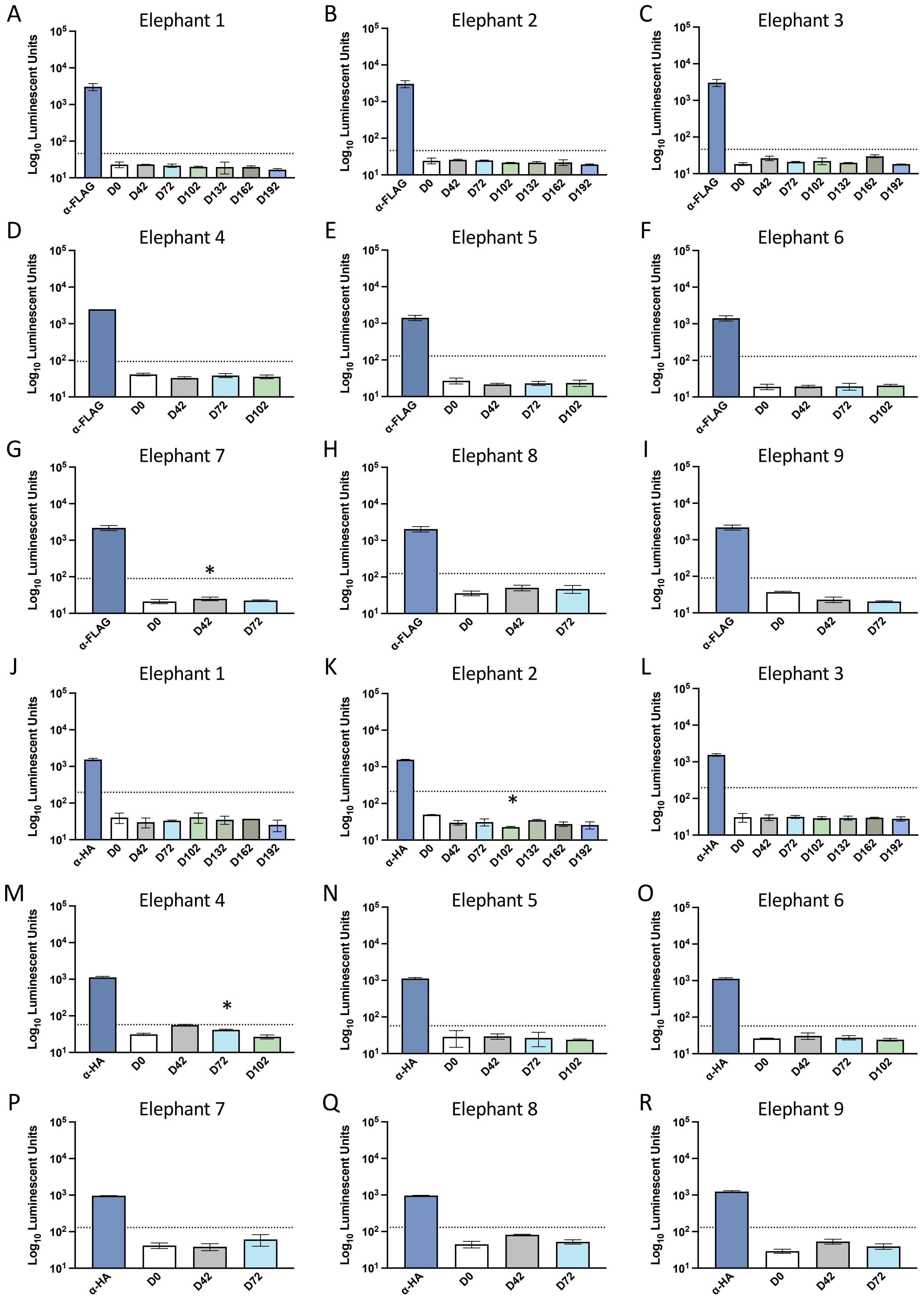

A
Elephant 1
B
Elephant 2
C
Elephant 3
D
Elephant 4
E
Elephant 5
F
Elephant 6
G
Elephant 7
H
Elephant 8
I
Elephant 9
J
Elephant 1
K
Elephant 2
L
Elephant 3
M
Elephant 4
N
Elephant 5
O
Elephant 6
P
Elephant 7
Q
Elephant 8
R
Elephant 9
Log10 Luminescent Units
α-FLAG
α-HA
D0
D42
D72
D102
D132
D162
D192
*

Figure legends

Figure S1. Determination of antibodies against EEHV1A ORFQ Clade A. Detection of anti-ORFQ Clade A antibodies by LIPS assay was measured for Elephants 1-9 for all timepoints presented in this study (A-I). Results are shown as luminescent units plotted on a $\log_{10}$ scale. Each bar represents the average of 2 technical replicates and is representative of two to three independent experiments. The dotted line represents the limit of detection determined by the mean of negative controls plus 5 standard deviations.

Figure S2. Determination of antibodies against EEHV1A ORFQ Clade C. Detection of anti-ORFQ Clade C antibodies by LIPS assay was measured for Elephants 1-9 for all timepoints presented in this study (A-I). Results are shown as luminescent units plotted on a $\log_{10}$ scale. Each bar represents the average of 2 technical replicates and is representative of two to three independent experiments. The dotted line represents the limit of detection determined by the mean of negative controls plus 5 standard deviations. Statistical significance was determined by One-way repeated measures ANOVA with Dunnett's test.

Figure S3. Determination of antibodies against EEHV1A ORFQ Clade D. Detection of anti-ORFQ Clade D antibodies by LIPS assay was measured for Elephants 1-9 for all timepoints presented in this study (A-I). Results are shown as luminescent units plotted on a $\log_{10}$ scale. Each bar represents the average of 2 technical replicates and is representative of two independent experiments. The dotted line represents the limit of detection determined by the mean of negative controls plus 5 standard deviations. Statistical significance was determined by One-way repeated measures ANOVA with Dunnett's test.

Figure S4. Linear range determination for LIPS and ELISA. Seropositive elephant serum was diluted 1:10 to 1:56250 in a 5-fold dilution series for LIPS (A-G) and 1:25 to 1:12800 in a 2-fold dilution series for ELISA (H). Simple linear regression was applied to results for at least three consecutive data points to obtain the R^2 value.

Figure S5. Recombinant protein production and purification workflow. Plasmids encoding gH, gL, and gO were transfected into HEK293 expi cells. The supernatant was collected four days after transfection. After a freeze/thaw cycle, suspensions were homogenized, treated for nucleic acid degradation, and further homogenized by syringe treatment. Samples were then clarified and filtered before affinity and size exclusion chromatography.

Figure S6. Characterization of EEHV1A gH/gL/gO recombinant protein. Chromatogram following SEC of gH/gL/gO on Superdex 200 column (A) demonstrating a single major peak. A Coomassie-stained SDS-PAGE gel with increasing quantities of purified gH/gL/gO in lanes 1, 3, and 5 separated by a marker in lanes 2 and 4 (B). Immunoblots of the gH/L/O complex run on an SDS-PAGE gel under non-reduced (C) and reduced (D) conditions and individual proteins detected by antibodies against their respective epitope tags for gH (StrepII), gL (Myc), and gO (FLAG).

Figure S7. Thermal stability of purified EEHV1A gH/gL/gO. Three replicate DSF melt curves show the thermal denaturation of 0.5 mg/ml gH/gL/gO in 25 mM HEPES (pH 7.5), 150 mM NaCl in the presence of 25X Sypro Orange demonstrating a Tm of 64.7 ± 0.3 °C.

Figure S8. Secondary structure analysis of purified EEHV1A gH/gL/gO by microfluidic modulation spectroscopy (MMS). Differential absorption profile with gaussian curve fitting determined signal contributions of distinct secondary structures from unordered regions (A). Calculated percentage of secondary structures and disordered regions (B).

Figure S9. Size-exclusion chromatography coupled with multi-angle light scattering (SEC-MALS) analysis of EEHV1A gH/gL/gO. Chromatogram showing the refractive index with calculated molecular weights. Total protein mass correlates to the theoretical mass of the gH/gL/gO trimer with a stoichiometry of 1:1:1.

Figure S10. Antibody response to multi-antigen EEHV1A mRNA vaccine in seropositive elephants. Detection of anti-gB (A-C), -gH (D-F), -gL (G-I), and -gO (J-L) antibodies by LIPS assay was assessed prior to vaccination through 192 through after. Results are shown as luminescent units plotted on a $\log_{10}$ scale. Each bar represents the average of 2 technical replicates and is representative of at least two independent experiments. The dotted line represents the limit of detection determined by the mean of negative controls plus 5 standard deviations. Antibodies against gH, gL, and gO were collectively assessed by indirect ELISA where each data point represents the average of 2 technical replicates from independent experiments (M-O). Results are depicted as ∆OD (signal from uncoated well subtracted from signal in a coated well). Statistical significance was determined by One-way repeated measures ANOVA with Dunnett's test.

Figure S11. Antibody response to multi-antigen EEHV1A mRNA vaccine in seronegative elephants (Group 2). Detection of anti-gB (A-C), -gH (D-F), -gL (G-I), and -gO (J-L) antibodies by LIPS assay was assessed prior to vaccination through 102 days after. The dotted line represents the limit of detection determined by the mean of negative controls plus 5 standard deviations. Antibodies against gH, gL, and gO were collectively assessed by indirect ELISA (M-O). Each data point represents the average of 2 technical replicates from independent experiments. Statistical significance was determined by by One-way repeated measures ANOVA with Dunnett's test.

Figure S12. Antibody response to multi-antigen EEHV1A mRNA vaccine in seronegative elephants (Group 3). Detection of anti-gB (A-C), -gH (D-F), -gL (G-I), and -gO (J-L) antibodies by LIPS assay was assessed prior to vaccination through 72 days after. The dotted line represents the limit of detection determined by the mean plus 5 standard deviations of the negative control. Antibodies against gH, gL, and gO were collectively assessed by indirect ELISA (M-O). Each data point represents the average of 2 technical replicates from independent

experiments. Statistical significance was determined by One-way repeated measures ANOVA with Dunnett’s test.

Figure S13. Two doses of EEHV1A multi-antigen mRNA vaccine do not induce significant antibodies against FLAG or HA protein tags. Detection of anti-FLAG (A-I) and anti-HA (J-R) antibodies by LIPS assay using *Guassia* luciferase fusion proteins was assessed over the course of the study. Results are shown as luminescent units plotted on a $\log_{10}$ scale. Each bar represents the average of 2 technical replicates and is representative of at least two independent experiments. A monoclonal antibody against FLAG or HA tag was used as a positive control and diluted 1:500 or 1:2000, respectively. The dotted line represents the limit of detection determined by the mean of the negative controls plus 5 standard deviations. Statistical significance was determined by one-way repeated measures ANOVA with Dunnett’s test. * $p<.05$.
